# Comparison of Whole-Body Electromyostimulation versus Recognized Back-Strengthening Exercise Training on Chronic Nonspecific Low Back Pain: A Randomized Controlled Study

**DOI:** 10.1155/2019/5745409

**Published:** 2019-09-29

**Authors:** Anja Weissenfels, Nicolas Wirtz, Ulrike Dörmann, Heinz Kleinöder, Lars Donath, Matthias Kohl, Michael Fröhlich, Simon von Stengel, Wolfgang Kemmler

**Affiliations:** ^1^Institute of Medical Physics, Friedrich-Alexander University of Erlangen-Nürnberg, 91052 Erlangen, Germany; ^2^Institute of Training Science and Sport Informatics, German Sport University Cologne, 50933 Köln, Germany; ^3^Department of Medical and Life Sciences, University of Furtwangen, 78054 Villingen-Schwenningen, Germany; ^4^Department of Sports Science, University of Kaiserslautern, 67663 Kaiserslautern, Germany

## Abstract

**Background:**

Low back pain (LBP) affects almost everyone at least once in their lifetime. Various meta-analyses show promising effects on pain reduction for conventional exercise. However, the lack of time and, especially for pain patients, a fear of movement (“kinesiophobia”) as well as functional limitations often oppose participation in such activities. In contrast, the advantage of novel training technologies like whole-body electromyostimulation (WB-EMS) lies particularly in a joint-friendly, time-effective, and highly customized training protocol and might be an alternative option for LBP patients. A meta-analysis of individual patient data and a comparison of WB-EMS against a passive control group confirmed the proof principle. Thus, the aim of this randomized controlled trial is to compare WB-EMS with a recognized back-strengthening exercise protocol to determine the corresponding effects on chronic, nonspecific LBP in people suffering from this.

**Methods and Findings:**

This randomized, controlled multicenter study is focused on novel and time-effective training technologies and LBP. In this contribution, the focus is primarily on the comparison of WB-EMS against a comparable conventional exercise training (CT). One hundred ten nonspecific chronic LBP patients, 40–70 years old, were randomly allocated to the intervention arms (WB-EMS: 55 vs. CT: 55). Both groups completed a 12-week program (WB-EMS: 1 × 20 min/week vs. CT: 1 × 45 min/week) specifically dedicated to LBP. The selection of the content of the active control group was based on the principles of WB-EMS training, which uses electrical stimulation to train mainly strength and stabilization in a very short time. Exercises were similar in all groups, with the focus on strengthening and stabilizing the trunk. Outcome measures were assessed by a four-week pain diary (before and during the last four weeks of intervention) as well as an isometric maximum strength measurement of the trunk muscles at baseline and after 12 weeks of intervention. Primary study endpoint was average pain intensity at the lumbar spine. Secondary study endpoints were maximum isometric strength of the back and the abdominals. The mean pain intensity of LBP decreased significantly in both groups (WB-EMS: −22.3 ± 20.9% vs. CT: −30.2 ± 43.9%; *p* < 0.001), however, without significant intergroup difference (*p*=0.160). A similar result was observed for “maximum isometric strength of trunk muscles.” The increase in back strength (WB-EMS: 15.6 ± 24.9% vs. CT: 23.0 ± 30.9%) was highly significant in both groups (*p*=0.001), and similar changes were observed for the trunk flexors (WB-EMS: 17.6 ± 24.8% vs. CT: 18.1 ± 24.8%). Also, at the secondary endpoint, no significant difference in pairwise comparison was observed in both cases (extension: *p*=0.297; flexion: *p*=0.707).

**Conclusion:**

In summary, both, WB-EMS and conventional back-strengthening protocol are comparably effective in reducing nonspecific chronic LBP in this dedicated cohort. The result is particularly positive in terms of time effectiveness and offers an adequate alternative for people with limited time resources or other barriers to conventional training methods.

## 1. Introduction

Due to changing living and working conditions [1, 2], the incidence of nonspecific low back pain (LBP) is increasing continuously worldwide [3]. The actual point prevalence (32–40%) and lifetime prevalence (74–85%) of LBP are very high [4]. However, LBP is not only a major individual health problem but also has an economic impact due to temporary or permanent inability to work and production losses [5]. Thus, more effort should be devoted to finding effective therapies that best meet current individual needs. Indeed, although present conservative therapies (e.g., resistance training, stabilization exercises, flexibility training, and aerobic exercises) demonstrate positive effects on LBP [6–8], some important limitations might prevent or at least aggravate the overall application in people with LBP. One aspect is the lack of time, which limits participation in time-consuming conventional exercise concepts [9]. In addition, many patients with LBP develop “kinesiophobia,” i.e., a fear of movement due to the inherent pain and are not willing to start a traditional dynamic exercise program [10]. Lastly, the observation in western societies that the majority of people are unable or unwilling to exercise frequently [11] requires the examination of optional training methods and technologies in the context of chronic unspecific LBP.

Due to their time-effectiveness, joint-friendly, and low (voluntary) loading characteristics, novel exercise technologies like whole-body electromyostimulation (WB-EMS) might be an option to time-consuming and intense back-strengthening protocols. Addressing effectiveness, the results of a recent meta-analysis on WB-EMS trials [12] summarized significant effects on LBP. The positive effect of electrical whole-body stimulation on LBP was also confirmed in a previous study between WB-EMS and a passive control group [13].

Under the premise that we expected the abovementioned exercise protocol to be effective in significantly impacting LBP, the aim of this study is to compare the effects of WB-EMS with a recognized back exercise program in people with nonspecific chronic LBP. There are many effective ways to reduce LBP, but we had to find a comparable form that would match not only the content context of WB-EMS (strengthening; stabilization) but also the time-effective character with one training session per week. Although a rough approach, the results of a meta-analysis [6] comparing conservative exercise methods (standardized mean difference (SMD): 0.50) provided less favorable changes in LBP compared with the WB-EMS specific meta-analysis of Kemmler et al. [12] (SMD: 0.84). Based on this rationale, our primary hypothesis is that WB-EMS is more effective in pain reduction than the conservative training method (CT). Our secondary hypothesis is that WB-EMS shows higher trunk strength gains than CT.

## 2. Materials and Methods

### 2.1. Trial Design

After an initial comparison of WB-EMS with a nonactive control group [13], this randomized, controlled trial in a parallel group design examines the next part of the multicenter study of the Institute of Medical Physics (IMP), Friedrich-Alexander University Erlangen-Nürnberg (FAU), and the German Sport University Cologne (DSHS), both in Germany. The focus here is on the comparison of WB-EMS with a recognized strengthening method in patients with chronic nonspecific LBP. Due to the interrelated character of the multicenter study, the following part shows similarities with the publication of the first implementation of Weissenfels et al. [13]. The study conformed with the Helsinki Declaration “Ethical Principles for Medical Research Involving Human Subjects” and was verified by the Ethical Committee of the FAU (ethics application no. 224_15b). The project was fully registered in the German Clinical Trial Register (impact of alternative exercise technologies on chronic low back pain in back pain patients under special regard of its sustainability; DRKS00009528). All study participants gave written consent to do the testing and interventions. The study reporting is based on the CONSORT 2010 guideline for randomized studies with a parallel group design [14].

#### 2.1.1. Participants

Participants between 40 and 70 years old were recruited by personal letters, which included the most important information as well as inclusion and exclusion criteria. In order to take part in the study, the following criteria had to be met: (a) 40–70 years old; (b) chronic pain in the lumbar spine (at least 50 percent of the days of the last three months); (c) no orthopedic diagnosis (unspecific type of LBP); (d) average basal pain intensity (numbering rating scale—NRS) ≥1); (e) no frequent intake of analgesics (>4 days/week); (f) no pharmacological therapy or diseases affecting muscle metabolism (e.g., glucocorticoids); (g) no contraindications for WB-EMS application (e.g., epilepsy, cardiac pacemaker, thrombosis, and total endoprosthesis); and (h) attendance in at least 10 of 12 units. A total of 12,000 letters were sent to which 650 persons responded. After checking the eligibility of the study, 110 participants were assigned to two intervention groups: WB-EMS (*n* = 55) and conventional training (CT) (*n* = 55). The assignment was randomized and stratified according to basal pain intensity (NRS: 1–3, 4–6, 7–10). In Table 1, the baseline characteristics of the subjects of all groups can be seen. The pain history of the participants showed that the majority of the cohort performed sitting or standing working activities (predominantly sitting: 41.8%; predominantly standing 40.0%), while only a small proportion performed changing (15.4%) or heavy physical work (2.8%). Their LBP, however, only leads in 9.1% of the cases to inability to work. Looking at the temporal history of the disease in 61.8% of the cases, the beginning of LBP was more than 5 years ago, in 21.8% between 2 and 5 years. The remaining participants reported that they suffered LBP between 6 months and 2 years.

### 2.2. Intervention

The interventions described below took place over a period of 12 weeks and were supervised by certified instructors. To ensure that effects were not influenced by other factors, participants were instructed to maintain their usual lifestyle during the study.

#### 2.2.1. Whole-Body Electromyostimulation (WB-EMS)

**Figure 1:**
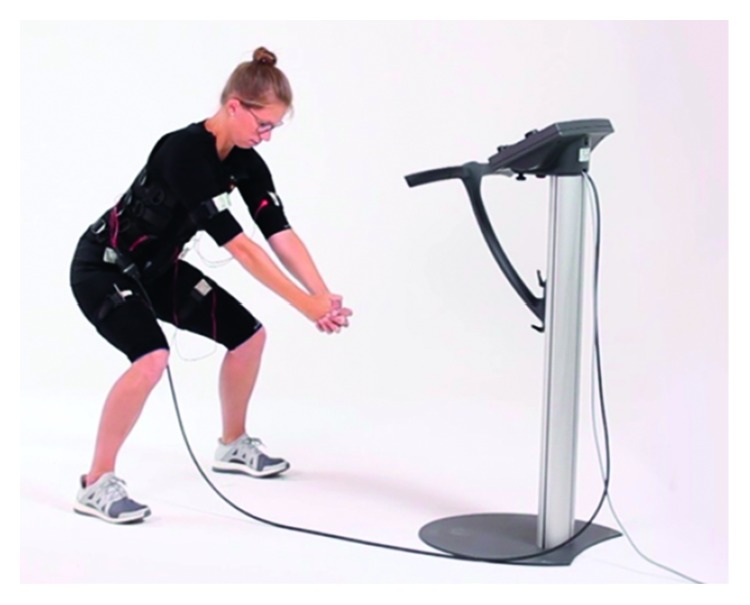
Alternative training technology whole-body electromyostimulation (WB-EMS).

WB-EMS is an innovative training technology that activates muscle contractions by electrical impulses. In comparison to the local version (e.g., TENS), WB-EMS addresses more muscle groups at the same time so that the most important parts are covered. Individual adjustment of the intensity is possible via separate control buttons. More details about this type of training can be found in several studies by Kemmler et al. [15–17]. We applied a common stimulation protocol (see Table 2) that is also used in commercial facilities. The main structure of the training was formed by exercises specifically dedicated to LBP (see Figure 1; see Table 2). Hence, no further joints were stressed, and participants performed all exercises with low amplitude. The WB-EMS training is based on guidelines from miha bodytec (Gersthofen, Germany), and therefore, a maximum of two individuals were trained once a week at the same time per instructor [18].

An objective control of the stimulation intensity is not possible with this type of training. Due to influencing factors, such as individual pain sensation, body composition, or humidity of the electrodes, the intensity can only be subjectively assessed by the participants. For this purpose, the BORG CR 10 scale was used, which converts the subjective feeling of intensity into values between 0 “nothing at all” and 10 “extremely strong/maximal” [19]. With exception of the first unit, participants were instructed to train at a rate of perceived exertion (RPE) between “strong (5)” and “very strong (7).” After a short time, the organism gets used to the selected stimulation intensity so that a continuous adjustment of the intensity has to take place during each session in close cooperation with the participants. Exercises with electrical stimulation can be very stressful for the organism, which is why the guidelines prescribe a habituation phase of several weeks [18]. During this phase, qualified instructors increase training duration weekly from 12 minutes to the final 20 minutes.

#### 2.2.2. Conventional Training (CT)

The CT program was based on exercises dedicated to back strength/core stabilization described by various meta-analyses [6, 7]. The decision for a conventional back-strengthening training is based on the comparability with WB-EMS, which averts strengthening and stabilization via electrical impulses. Each weekly session took 45 min: 15 min of aerobic warm up and 30 min circle training. The selection of the exercises is according to the stimulation areas of WB-EMS and so we focused on dynamic and static exercises for the trunk (see Table 3). Detailed training content and exercise parameters can be found in Table 3. In order to compare the intensity between both intervention groups, the participants were also instructed to perform exercise level between “strong (5)” and “very strong (7)” on the BORG CR 10 scale. During each session, a qualified instructor observed and corrected the participants' movement execution. A standardization of the training was granted by audible support and announcements of the working and resting times as well as station descriptions on cards.

### 2.3. Outcomes

 Primary endpoint
  (i) Changes in average low back pain intensity from baseline to 12-week follow-up
 Secondary endpoint
  (i) Changes in maximum isometric trunk extension from baseline to 12-week follow-up
  (ii) Changes in maximum isometric trunk flexion from baseline to 12-week follow-up

### 2.4. Assessments

All tests were performed using the same procedure and by the same researcher in each of the three study periods. Baseline and follow-up tests took place at a similar time of day (±60 min). To ensure proper standardization, participants were requested to avoid severe physical activity 24 h prior to the assessments. They were also asked to fast for 3 h prior to the measurements.

All parameters for anthropometry were determined with calibrated devices. To determine basal values and morphological changes, body height was measured barefoot via stadiometer. A Bio-Impedance Analysis (DSM-BIA, InBody 770, Seoul, Korea) detected weight and body composition. Based on the electrical conductance of tissue, the device can measure the distribution of body fat mass and muscle mass in different segments (arms, trunk, and legs). In this case, it operates with six frequency ranges between 1 and 1000 Hz.

The primary endpoint LBP intensity was protocolled through a four-week pain diary prior to the intervention and in the last four weeks of training. A numerical rating scale (NRS) from 0 (no pain) to 10 (worst possible pain) was used to indicate the daily pain intensity [20]. In addition to LBP intensity, the duration of pain, special features, and the intake of medication were also examined. A dedicated questionnaire included other tools for back pain research (German pain questionnaire, chronic pain grade (GCPS), and Roland and Morris Disability Questionnaire (RMDQ)) [21–23]. To check influencing factors, baseline and follow-up questionnaires included questions about diseases, medication, and lifestyle (changes).

For the functional measurement of the isometric trunk strength, a Back-Check 607 (Dr. Wolff, Arnsberg, Germany) was used. Depending on trunk flexion and extension, the device is positioned differently and the recommendations of the manufacture were observed. In both types of testing, all participants were fixed at the level of iliac crest in an upright position (0°) with angled knees (20°). To measure the maximum trunk flexion strength (i.e., abdominal strength), a measuring electrode was positioned at the level of the sternum, while the maximum trunk extensor strength (i.e., back strength) was measured at scapula level. Based on the tree test rounds, the highest value was included in the analysis. In order to comply with quality criteria, reliability (test-retest reliability; intraclass correlation (ICC)) for maximum trunk extension in this cohort was 0.88 (95% CI: 0.82–0.93), whereas the value for maximum trunk flexion was slightly lower at 0.86 (95% CI: 0.81–0.90) [13].

#### 2.4.1. Sample Size, Randomization, and Blinding

The calculation of the sample size is based on the results of a current meta-analysis by Searle et al. [6] that examines the effects of conventional types of exercise on LBP and on data from a meta-analysis of individual patient data [6, 12]. Based on these studies, we supposed a SMD of 0.55 for the primary hypothesis using the 0–10 NRS. Addressing the core study hypothesis by a *t*-test (i.e., differences between the WB-EMS and the CT), 54 participants per group were required to generate *α* = 0.05 and *β* − 1 = 0.80 (80% power).

**Figure 2:**
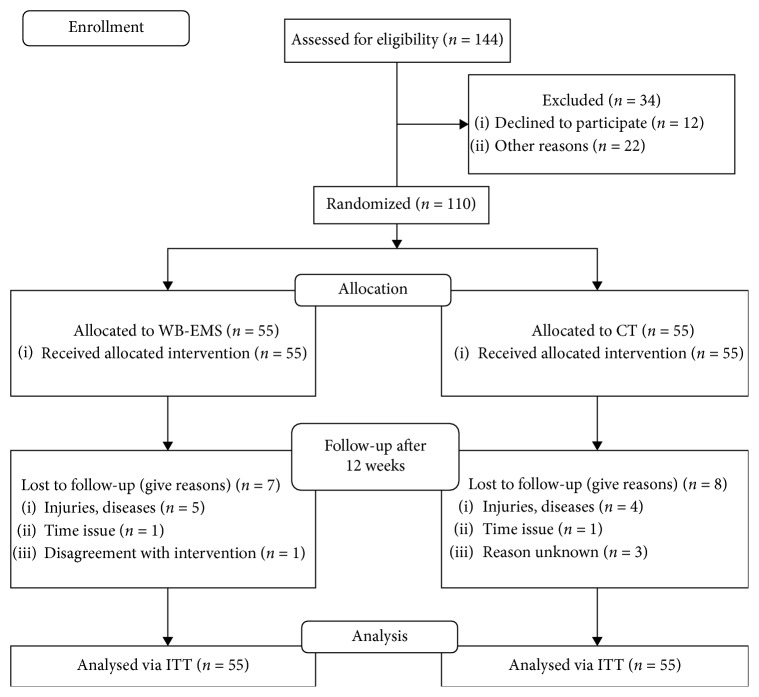
CONSORT flow diagram of the study intervention.

In three conservative rounds (April 2017 to August 2018), a total number of 110 participants were balanced (1-1) and randomly assigned into two equal groups by drawing lots themselves (see Figure 2). These were placed in opaque plastic shells and stratified according to the NRS (0–3, 4–7, 7–10). In this context, it should be noted that neither participants nor researchers were able to know the allocation beforehand. Thus, the guidelines for allocation concealment were realized consistent. While 30 participants (WB-EMS: 15 vs. CT: 15) were trained in the first round, the number of persons in the following two rounds increased to 40 per round (WB-EMS: 20 vs. CT: 20). After each balanced group allocation (in total 55 participants per group), participants were informed about the further study process and asked not to change their usual lifestyle.

Due to the participation in different kinds of exercise programs and for organizational reasons, a blinding of the study participants, instructors, and primary researchers is generally not possible for this kind of studies. Therefore, we concentrated on the blinding of the research assistants/outcome assessors so that no correlation of the group status was possible. In this case, the blinding was only partial.

### 2.5. Statistical Analyses

For the primary and secondary endpoints, an intention-to-treat (ITT) analysis was used, which included all participants no matter of compliance or lost to follow-up. This type of analysis represents the “golden standard” for clinical randomized trials (RCTs) [24]. The calculation was done with *R* statistics software in combination with multiple imputation by Amelia II [25]. In this process, the entire data set was applied for multiple imputation, with imputation being repeated 100 times. A dependent *t*-test was conducted to analyze within-group differences. For this purpose, primary and secondary endpoints were statistically (Shapiro–Wilk test) and graphically (QQ and box plots) checked for normal distribution before. Independently of the endpoints mentioned, a Welch *t*-test was used to calculate pairwise intergroup differences [26]. SPSS 25.0 (SPSS Inc, Chicago, IL) was selected for the statistical calculation of baseline data, and all results were presented as mean value (MV) and standard deviation (SD). As usual, a statistical significance of *p* < 0.05 was assumed for the analysis.

## 3. Results

Fifteen participants dropped out after the start of the study for various reasons: (1) injuries, diseases (*n* = 9); (2) time constraint (*n* = 2); (3) disagreement with intervention (*n* = 1); (5) reason unknown (*n* = 3). For the dropout rate no significance between the groups was measured (*p* < 0.784). However, due to our ITT approach with missing data imputation, 55 participants per group were included in the analysis. Except for age, no significant differences were observed for baseline characteristics between WB-EMS and CT group (see Table 1).

Attendance rate was high for both groups (WB-EMS: 92.0 ± 7.4%; CT: 87.2 ± 8.5%), with a significant intergroup difference (*p* < 0.004). All participants reported having performed the intervention exactly according to the study protocol. With the exception of one participant in the WB-EMS group, no adverse or unintended side effects were observed during the training sessions, and no participants reported any WB-EMS or CT-related discomfort during or after application/intervention. This person had to interrupt the intervention due to gastritis, which was not caused by WB-EMS.

The results of the primary study endpoint include a highly significant decline in average pain intensity (WB-EMS: −22.3 ± 20.9%; CT: −30.2 ± 43.9%; *p* ≤ 0.001), however, without a significant intergroup difference (*p*=0.160) (see Table 4). Thus, we have to revise our hypothesis that WB-EMS shows better changes of average low back pain intensity than in CT.

Maximum isometric trunk extensor strength significantly increased (*p* ≤ 0.001) in both study groups by 15.6 ± 24.9% (WB-EMS) and 23.0 ± 30.9% (CT), respectively. Similar changes were observed for the trunk flexors (WB-EMS: 17.6 ± 24.8%; CT: 18.1 ± 24.8%). Abdominal maximum isometric strength also significantly increased (*p* ≤ 0.001); however, again we failed to detect significant differences between the groups (extension: *p*=0.297; flexion: *p*=0.707). Thus, we revised our secondary hypothesis that WB-EMS shows significantly better developments of maximum isometric trunk strength compared with CT.

With regard to lifestyle changes affecting LBP, additional treatments were prohibited during the intervention. In total, 12 participants (WB-EMS: 4; CT: 8) started an additional treatment during intervention, which mainly included massage, physiotherapy, acupuncture, osteopathy, or sports at work. On the other hand, four subjects stopped a previously started treatment: WB-EMS: 1; CT: 3. The number of participants with acute intake of analgesics also changed. In the WB-EMS group, it decreased from 15 to 9 persons and in CT group from 17 to 8 persons. With regard to the parameters mentioned above, there are no significant differences between the groups (see Table 5).

## 4. Discussion

The aim of this study was to compare the effects of the novel training technology WB-EMS on chronic low back pain with a recognized back training program in a mixed cohort of men and women between 40 and 70 years with nonspecific chronic LBP. In summary, the results for average pain reduction and increase of muscle strength in trunk are highly effective in both groups, however, without any significant intergroup differences.

To our best knowledge to date, there is currently no other trial that compares WB-EMS with a conventional method with regard to the effect on nonspecific chronic LBP. In contrast to the large amount of studies that focus on the effect of conventional exercise on chronic LBP [6–8], there are hardly any studies that focus on the effect of WB-EMS on LBP. Apart from a published masters thesis [27], which evaluates the effect of WB-EMS on LBP rather than an experimental endpoint in a healthy clientele, only a meta-analysis of individual patient data [12] addresses this issue. In summary, after WB-EMS interventions ranging from 14 weeks to 12 months, pain intensity of lower back decreased by 16.9% on a 7-level scale [12]. These results are very similar to the current study, while age (55.2 ± 7.7 vs. 72.0 ± 7.1 years), higher training frequency (1.0 vs. 1.5 sessions/week), and LBP-specific vs. unspecific assessment tools might explain the slight difference between the present study and the results of the meta-analysis.

Although several studies (e.g., [28–30]) demonstrated the efficacy of conservative methods in the therapy of LBP, some of the concepts applied differ considerably from the design of the current trial and are therefore hardly comparable. Close to our study, Yang and Seo focused on conservative exercise, especially, stabilization of the trunk, in patients with nonspecific chronic LBP [31]. After 6 weeks of intervention (3 session/week; 30 min per session), this group achieved a pain reduction of 33.3%, measured via VAS. Another study that applied a lumbar stabilization program with 106 middle-aged workers for one year reported even higher pain reduction rates of 44% that also determined via VAS (7 days; 4 weeks) [32]. However, compared with the present study, the training frequency was higher (1.0 vs. 2.0 sessions/week) and the intervention was conducted over a much longer period of time (12 weeks vs. 12 month). Of interest, the results after 6 months were much lower (VAS past 7 days: −34.3%; VAS past 2 months: −22.2%), which imply that the length of the intervention might be a relevant predictor for the amount of pain reduction. Consequently, we speculate that longer study periods will result in more pronounced effects of pain reduction.

Summarizing the results of the secondary study endpoint “maximum isometric trunk strength,” no significant intergroup difference could be measured; however, the increase in strength was highly significant across both groups. Only a few studies verify this endpoint in patients with LBP; their assessment methods differ greatly. Therefore, a comparative discussion of our results with the present literature is very limited. As to our best knowledge, this is the first evidence-based study on WB-EMS and LBP, and we have to refer to comparative studies with other clients or diseases. Only one study [33] including elderly women (75 ± 4 years) with sarcopenia reviewed the change of maximum isometric trunk extension after a WB-EMS intervention (1.5 sessions/week; bipolar; 6 sec load—4 sec break; 85 Hz). After 12 months, maximum isometric trunk extensor strength increased significantly (*p* ≤ 0.001) by 10.1 ± 12.7%. Despite a longer intervention phase, the results of lumbar strength are lower than in the present trial; however, the clientele of the study of Kemmler et al. [33] was much older and suffered from diseases that might confound the proper effect of WB-EMS on muscle (strength). Most important, however, the additionally performed movement patterns during WB-EMS did not consistently focus on abdominal and back strength during the latter study.

Studies focusing isolated lumbar extension resistance training [28, 30] and particularly those that train on the dedicated devices (e.g., MedX, Gainesville, FL) reported almost twice as high strength gains compared with our more conventional strengthening exercise program. Less specific, Moon et al. [34], who compared two lumbar exercise protocols (isometric vs. dynamic), determined lumbar extension strength at different points of flexion angle (0°–72°) using a specified assessment device (Med X, Gainesville, FL). After 8 weeks of intervention (2 sessions/week; 60 min in total; 14–16 exercises), maximum isometric extension strength (0° position) increased by 30.0% in the dynamic and 48.5% in the isometric study group, with a significant intergroup difference (*p* ≤ 0.05) on this angle point [34].

In summary, independent of the exercise group, changes in primary and secondary endpoints of the present trial are very satisfying and range in the (upper) area of existing studies. Since there are no comparable studies using this design, it is not possible to discuss differences between the active groups in more depth. Our finding of the favorable effects of WB-EMS on chronic nonspecific LBP should further result in a revision of the “negative recommendations” for electrotherapy according to the (German) National Guideline for Back Pain [35].

Despite the current lack of evidence for a superiority to conventional LBP programs, a major advantage of WB-EMS is the time efficiency. Considering the net workout time, we determined a highly significant difference between the groups (WB-EMS: 200.1 ± 22.6 min vs. CT: 471.1 ± 45.8 min; *p*=0.001). In other words, WB-EMS generated similar effects on LBP as CT but in less time. Considering that time constitutes a rather limited resource for many people, this aspect might be the most striking argument for the application of novel exercise technologies.

However, some limitations might decrease the scientific evidence of our results. (1) One may argue that the comparison of two different back-specific training methods always entails limitations, but based on the aim to verify options for conventional back exercises, the study represents a realistic situation. Due to the strengthening and stabilizing content of WB-EMS, the decision has been made in favor of a back-specific-strengthening circle. In addition, similar exercises were performed and comparable muscle groups were trained. However, a comparison of identical protocols with and without electrical stimulation is not effective. On the one hand, this is because of the low intensity of the exercises without electrical stimulation, low ROM, and low loading time, which will not bring any benefit to the patients and, on the other hand, to create a training situation which is not related to real situations. In this sense, the comparison of WB-EMS with CT should represent realistic and effective training measures. (2) Another limitation that might influence the WB-EMS results is the low level of strain intensity applied. Although the average intensity of RPE 5.8 ± 1.1 reported by the participants was within our prescribed target range (5 “hard” to 7 “very hard”), it is lower than in most other WB-EMS trials [36]. We speculate that generating higher strain intensities will result in higher effects in LBP and particularly in strength changes; however, it is difficult to implement this in a pain-sensitive cohort. (3) As addressed above, a further reason that might lower the results of this trial below comparative studies might be the rather low inclusion criterion of NRS ≥ 1 that resulted in average baseline pain intensity levels of 2.8 ± 1.5. This, however, is considerably lower than in comparable studies. For comparison, Yang and Seo [31] listed even 5.4 ± 1.4 on a VAS 0–10 scale and other studies that used 0–100 VAS scales observed average basal pain level of 34 ± 18 [34] to 40 ± 4 [37]. Under the premise that higher baseline values should allow more pronounced exercise-induced reductions in low back pain, our strategy to include subjects with lower levels of unspecific chronic low back pain was less constructive. Correspondingly, using baseline level of pain parameters as inclusion criterion for further studies should be very carefully considered. (4) Based on the different intervention time protocols per week (WB-EMS: 20 min vs. CT: 45 min), the study may be said to have been arbitrarily designed to advantage one of the groups. However, the trial was adjusted as close as possible to reality using the current settings used on the healthcare market. (5) Of course, the generalization of our results to the entire cohort of people with LBP is limited. In this study, we determined a positive effect of WB-EMS and CT on chronic, nonspecific LBP in people between 40 and 70 years old, which constitutes the most relevant group of LBP patients [38]. However, it would inappropriate to directly transfer our results to (1) other age groups, (2) acute pain periods or conditions, or (3) specific types of back pain (e.g., vertebral fractures). With respect to the latter aspects, exercise interventions might be even detrimental and contraindicated at least in the early stage of acute trauma-induced LBP. Further, the intervention has to be much more customized when addressing specific orthopedic indications of LBP. However, although not reported here, we did not detect gender or age-dependent differences in the efficacy of WB-EMS or CT in our cohort. (6) Though the participants were consistently encouraged to maintain their habitual lifestyle, some participants reported changes with potential effects on our study endpoints. While 12 participants started a new therapy during intervention, four people stopped an existing treatment. Nevertheless, since the corresponding distribution within the study groups was quite similar, this bias should not have relevantly affected our study results.

## 5. Conclusion

In summary, both types of exercise, WB-EMS and CT, significantly reduce LBP intensity in people with chronic, nonspecific LBP, without relevant differences between the interventions. Although we have to revise our hypothesis of the corresponding superiority of the novel exercise technology WB-EMS, from a pragmatic point of view, these results are more than welcome. Depending on time availability, medical cofactors, and personal preferences, patients can individually decide which training suits them best. WB-EMS offers an alternative and expands the range of effective training options for LBP to those who cannot or do not want to perform a conventional back-strengthening exercise program. In addition, the results show that this alternative training technology, which was originally developed for the fitness sector, is absolutely relevant for the conventional treatment of clinical diseases such as LBP.

## Figures and Tables

**Table 1:** Baseline characteristics of both intervention groups.

Variable	CT (*n* = 55)	WB-EMS (*n* = 55)	*p*
Gender (m/f)^a^	17/38	20/35	0.549
Age (years)^a^	57.4 ± 7.6	54.4 ± 7.4	0.035
Height (m), m/f^b^	1.83 ± 4/1.66 ± 7	1.82 ± 5/1.67 ± 7	0.825/0.440
Weight (kg), m/f^c^	87.9 ± 7.5/73.5 ± 15.4	90.3 ± 15.2/73.9 ± 14.5	0.567/0.913
Total body fat (%), m/f^c^	23.4 ± 4.3/35.0 ± 8.2	25.1 ± 8.9/32.9 ± 8.7	0.485/0.304
RMDQ (number of items)^a,d^	4.8 ± 3.3	5.6 ± 3.9	0.277
Acute use of analgesics (*n*)^a^	17	15	0.585
No regular exercise (*n*)^a^	5	6	0.800

^a^Assessed by baseline questionnaire. ^b^Measured via stadiometer. ^c^Measured via Bio-Impedance Analysis (DSM-BIA, InBody 770, Seoul, Korea). ^d^RMDQ measured functional limitations due to low back pain and consists of a 24-point scale.

**Table 2:** Exercises of WB-EMS intervention.

Exercise sequence for the WB-EMS group
The total duration of a unit are 20 minutes, with a habituation phase of 4 weeks (12 to 20 min/unit). Each session contains of 6 trunk specific exercises with 3 sets a 6 repetitions with the usual stimulation parameters of WB-EMS (bipolar, 85 Hz, 350 *μ*s, 6 sec stimulation and 4 sec rest, 1 unit/week)
(1) Squat with latissimus pulleys
(2) Butterfly reverse (with angled arms)
(3) Straight pullovers with trunk flexion (lumberjacks)
(4) Standing trunk flexion (crunch)
(5) One-legged stand with biceps curl
(6) Side step with weight shift and biceps curl

**Table 3:** Exercises of conventional training group.

Exercise sequence for the CT group
In addition to a 15-minute warm up, the CT intervention is constructed like a circle with 10 trunk specific exercises. The circle is done twice with 50 sec work and 25 sec break between each exercise. Every three weeks, the exercises slightly changed so that the intensity is adjusted
(1) Seated rowing with cable pull
(2) Cable pulldown
(3) Crunch
(4) Plank
(5) Dynamic squat with arm movement
(6) Bird dog
(7) Side plank
(8) Static situp
(9) Back extensor
(10) Static hip bridge ⟶ dynamic hip bridge

**Table 4:** Results of the primary and secondary endpoint after 12 weeks of intervention.

	CT MV ± SD (*p*)	WB-EMS MV ± SD (*p*)	Absolute difference MV (95% CI)	*p*
Average pain intensity (4 weeks) (Index)^a^				
Baseline	2.81 ± 1.34	2.69 ± 1.52	—	0.689
Difference	−0.85 ± 0.97^*∗∗∗*^	−0.60 ± 0.96^*∗∗∗*^	0.25 (−0.10 to 0.60)	0.160
Maximum isometric trunk extension (kg)				
Baseline	38.90 ± 15.54	46.20 ± 19.13	—	0.062
Difference	8.96 ± 8.78^*∗∗∗*^	7.19 ± 8.82^*∗∗∗*^	1.77 (−1.58 to 5.12)	0.297
Maximum isometric trunk flexion (kg)				
Baseline	36.61 ± 17.05	41.47 ± 15.98	—	0.126
Difference	6.61 ± 9.09^*∗∗∗*^	7.30 ± 9.05^*∗∗∗*^	0.69 (−4.26 to 2.88)	0.707

^a^Index from 0 (no pain) to 10 (worst possible pain). ^*∗∗∗*^ *p* ≤ 0.001.

**Table 5:** Training characteristics and confounders with potential impact of the study endpoints.

Variable	CT *n* = 55	WB-EMS *n* = 55	*p*
Dropout rate (*n*)	8	7	0.784
Attendance (%)	87.2 ± 8.5	92.0 ± 7.4	0.004
Total training time (min)	471.1 ± 45.8	200.1 ± 22.6	0.001
Changes of acute intake of analgesics (*n*)	17 ⟶ 8	15 ⟶ 9	0.791

## Data Availability

Data are available upon request to the Institute of Medical Physics of Friedrich-Alexander University Erlangen-Nürnberg (anja.weissenfels@imp.uni-erlangen.de) for researchers who meet the criteria for access to confidential data.
